# Time trends and persistence in PM_2.5_ in 20 megacities: evidence for the time period 2018–2020

**DOI:** 10.1007/s11356-022-22512-z

**Published:** 2022-08-18

**Authors:** Lorenzo Bermejo, Luis A. Gil-Alana, Marta del Río

**Affiliations:** 1 Faculty of Economics, Universidad Villanueva, Madrid, Spain; 2 Department of Economics, Faculty of Economics, University of Navarra, E31008 Pamplona, Spain; 3 University Francisco de Vitoria, Madrid, Spain

**Keywords:** Particular matters, PM_2.5_, Long memory, Fractional integration, Time trends, C22, Q53

## Abstract

The degree of persistence in daily data for PM_2.5_ in 20 relevant megacities such as Bangkok, Beijing, Mumbai, Calcutta, Canton, Dhaka, Delhi, Jakarta, London, Los Angeles, Mexico City, Moscow, New York, Osaka. Paris, Sao Paulo, Seoul, Shanghai, Tientsin, and Tokyo is examined in this work. The analysis developed is based on fractional integration techniques. Specifically, the differentiation parameter is used to measure the degree of persistence in the series under study, which collects data on daily measurements carried out from January 1, 2018, to December 31, 2020. The results obtained show that the estimated values for the differentiation parameter are restricted to the interval (0, 1) in all cases, which allows us to conclude that there is a mean reverting pattern and, therefore, transitory effects of shocks.

## Introduction

Human health, and particularly for those who live in cities, can be seriously affected by poor air quality. Recent studies on health and the environment have pointed out that one of the most harmful pollutants for health is suspended particles, especially the finest particles. Fine particles, also called particulate matter or PM_2.5_, can penetrate deep into the lungs and cause them to become inflamed, putting patients with heart and lung disease in serious danger. In turn, these particles can carry carcinogenic compounds that could be adsorbed on the surface of the lungs. All of this leads research on the dynamics of atmospheric pollution to acquire great importance, specifically research projects that contribute and develop models for prediction purposes and, as a consequence, that enable the design of air quality management policies.

Based on the above, the present paper investigates the time series properties corresponding to daily data of PM_2.5_ on the twenty megacities around the world, investigating its evolution across time. We measure the degree of persistence to determine whether the shocks in the series have permanent or transitory effects. However, instead of using classical methods, which are based on a “well” I(0) or short-memory behaviour of the error term, we consider the possibility of long memory, which is a feature very often observed in environmental and climatological data (Liu et al. 2014; Yaya et al. 2015; Knight et al. 2017; Li et al. 2017; Bai et al. 2019; Qi et al. 2019; Zhao et al. 2019; Gil-Alana and Lenti 2021; Gil-Alana et al. 2020a, b; Sakiru et al. 2021; etc.).

The term “megacity” is used to define a metropolitan area with more than 10 million inhabitants. Typically, these urban environments are made up of one, two, or more metropolitan areas that have been physically joined together (Rollandi 2012). However, information from different sources differs on the number of inhabitants in megacities, mainly because the urban area is spread over territories that are divided by different political entities, and sometimes, there is no clear definition of urban boundaries. A characteristic of Megacities is that they are polycentric, which means that they do not have a single centre, but that within the same urban extension there are different areas with the capacity to attract economic, social, and political activities. In this case, megacities are structured with the existence of different centralities. There are major differences between megacities located in developed countries and those in developing countries. On the one hand, in developed countries, there are conurbations organised by the extension of infrastructures over territories that are being incorporated in an orderly fashion. However, in megacities in developing countries, conurbations are created through informal settlements, which have no planning or infrastructure. In fact, urban planning in these environments usually takes place after the consolidation of an urban area and is reduced to providing infrastructure for the new neighbourhood (Giglia 2001).

On these occasions, urban management focuses on what is urgent rather than what is important. Governments are often more concerned with finding solutions to specific, immediate, and emerging problems (flooding, rubbish, water supply, etc.) than with solving the pollution problems of an area in the medium or long term (Cantos 2011).

One of the most important reasons for the increase in environmental problems is population growth. In order to meet the needs of the population, industrial development is necessary. But increasing industrialisation causes rapid consumption of natural resources. In addition, the waste produced by production and consumption has a negative impact on the environment. Another negative factor in population growth is unplanned urbanisation. As a consequence of unplanned urbanisation, pollution is increasing in urban centres. Air, water, and soil are polluted, negatively affecting living beings.

It is clear that how megacities continue to produce and consume energy and goods will be crucial to their social, ecological, and economic survival. In this context, policies related to environmental sustainability will be key to hopefully facing one of the great challenges facing humanity.

The concentration of the world’s population in urban centres is a growing trend. According to the United Nations (UN), about half of the world’s population (55%) now lives in urban areas; and by 2050, about two-thirds (68%) of all people are projected to reside in urban areas (World Population Prospects 2019). The UN points out that while in 1990 there were 10 megacities in the world, today their number has risen to 33, and they have grown from 7% of the world’s population to 13%, a trend that will continue in the future (UN 2019).

Population growth in megacities is mainly determined by migration. The explanation lies largely in the migratory flow, in the transfer of population from other populations, which does not necessarily have to become international immigration, since a large part of the world’s population will move from rural to urban areas (UN 2021). In this context, we have taken 20 megacities with different levels of industrialisation and development as reference for our study, which are located in different regions worldwide, and for which reliable records on PM_2.5_ emissions are available. See Table 1 below that displays the population of these megacities in 2020.

**Table 1:** Population in the 20 megacities analysed in 2020

City	State	Population
Bangkok	Thailand	16.400.000
Beijing	China	21.895.000
Bombay	India	25.200.000
Calcutta	India	16.800.000
Canton	China	15.310.000
Dacca	Bangladesh	28. 399. 000
Delhi	India	30.300.000
Jakarta	Indonesia	29.200.000
London	UK	14.900.000
Los Angeles	USA	17.800.000
Mexico	Mexico	22.200.000
Moscow	Russia	19.400.000
New York	USA	22.100.000
Osaka	Japan	17.700.000
Paris	France	12.400.000
Sao Paulo	Brasil	22.200.000
Seoul	Korea	26.000.000
Shanghai	China	38.000000
Tientsin	China	12.000.000
Tokyo	Japan	38.400000

World Bank

The rest of the paper is structured as follows: The “A Review of the Literature” section shows a brief review of the literature on the topic. Data and the methodology and modelisation used in the paper are given in the third section. The data used are presented in the fourth section. The empirical results are displayed in the fifth section, while the last section concludes the paper.

## A review of the literature

Poor air quality can affect human health. Among the most harmful pollutants for health is particulate matter (PM_2.5_). In recent years, greater attention has been paid to particles with a size smaller than 1 μm in diameter, which are known as an ultrafine fraction and to which it seems can be attributed a greater potential for damage (Lippmann 1989; Ostro et al. 1999; Castillejos et al. 2000; EPA 2002; Morgenstern et al. 2007; Xing et al. 2016; Eguchi et al. 2018; Amsalu et al. 2019; etc.)**.**

Suspended particles or fine particles can enter the lungs, causing inflammation in the lungs and producing serious diseases. Suspended particles also often carry carcinogenic compounds that can be absorbed by the lungs. The main components of the particles are metals (lead, iron, vanadium, nickel, copper, platinum, and others), organic compounds, material of biological origin (viruses, bacteria, animal, and plant remains, such as pollen fragments), ions (sulphates, nitrate, and acidity) and reactive gases (ozone, peroxides, and aldehydes), and their core is often made up of pure elemental carbon (EPA 1999).

Air pollution is a global problem, affecting all countries regardless of their level of development. It can occur in indoor environments, where the main pollutant is tobacco, or in outdoor environments, in which case industry and massive vehicular load are the main associated factors. The rapid growth of cities coupled with the lack of effective transportation planning can be the cause of large and harmful levels of fine particulate matter (PM_2.5_) in the air (Montes de Oca et al. 2010).

Numerous institutions in different countries and several studies have analyzed the connection between pollution and adverse health effects. Examples are the works by Schwartz and Marcus (1990), Anderson et al. (1996), Atkinson et al. (1999), Gardner and Dorling (1999), HEI (2002), EPA (2002), and WHO (2006). In the USA, Section 812 of the Clean Air Act Amendments requires the Environmental Protection Agency (EPA) to periodically evaluate the effects of the Clean Air Act on public health, the economy, and the environment. In this document, a series of measures are proposed to improve air quality, as well as to establish a detailed program for compliance and maintenance of national air quality standards. In this sense, it is clearly important to investigate the dynamics of air pollution in order to develop adequate models for prediction purposes and to design policies to manage air quality.

In more developed cities, particulate pollution from car traffic is a major problem. In many cases, these cities have grown without proper planning of their growth in many areas, such as transport flow planning. The fact that we do not have frequent air quality data makes it difficult to assess health impacts and for governments, in some cases, to take policies on increasing transport flows seriously. In this respect, it is of vital importance to define an air quality standard in order to protect citizens (Cohen et al. 1997; Rosales-Castillo et al. 2001; Magas et al. 2007; Molnár et al. 2007; Montes de Oca et al. 2010; Yuan et al. 2012; Steinle et al. 2013, 2015; Karagulian et al. 2019).

In this regard, the World Health Organization (WHO 2006) has established an annual limit value of 10 μg/m^3^ for the concentration of PM_2.5_ particles in the air. However, in some large cities, this value practically doubles, with the consequent impact on morbidity and mortality. Recently, the WHO has established new guideline values for particulate matter concentrations in the air based on concentrations of particles smaller than 10 μm in diameter (PM_10_) and particles smaller than 2.5 μm in diameter (PM_2.5_), although it clarifies that PM_2.5_ values are preferable to PM_10_. This preference is based on the fact that PM_10_ has an important component of natural origin, especially in southern European cities, such as air intrusions from North Africa. (Rodríguez et al. 2001; Escudero et al. 2005). However, in an urban atmosphere, the main contribution to PM_2.5_ is due to engine combustion and has a less important natural component than PM_10_ (Ballester et al. 2007), and therefore seems, a priori, to be a more reliable indicator for measuring anthropogenic activity. In addition, these fine particles penetrate deeper into the pulmonary alveoli producing more adverse health effects than particles of a larger diameter, such as PM_10_ (De Kok et al. 2006). In another study, Linares and Díaz (2009) explain, for example, the association found between PM_2.5_ and children’s hospital admissions to the emergency room, compared to what had been detected with other pollutants. Similar evidence is also found in Bell et al. (2015), Patto et al. (2016), Nishikawa et al. (2021), and Ren et al. (2021) among many others.

In this article, we work with a long-memory perspective based on fractional integration. Long memory is an aspect of the time series where the high degree of dependence between indicators that are widely separated in time stands out. Analysing the bibliography on this methodology, we find that it has been used in many areas of knowledge, for example in the economic-financial field (Gil-Alana and Moreno 2012; Abritti et al. 2017; Kalemkerian and Sosa 2020; Murialdo et al. 2020; Qiu et al. 2020); in the tourism sector (Al-Shboul and Anwar 2017; Pérez-Rodríguez and Santana-Gallego 2020), as well as in climatology and environmental sciences (Gil-Alana 2005, 2008, 2017; Vyushin and Kushner 2009; Franzke 2012; Ludescher et al. 2016; Barros et al. 2016; Tiwari et al. 2016; Bunde 2017; Yuan et al. 2019; Gil-Alana and Solarin 2018; Gil-Alana and Trani 2019; Bruneau et al. 2020; Gil-Alana et al. 2020a, b; Xayasouk et al. 2020; Yaya et al. 2020). In this latter area, the most recent writing using fractional integration is that of Caporale et al. (2021). In the study, the authors investigate the statistical properties of daily PM_10_ in eight European capitals (Amsterdam, Berlin, Brussels, Helsinki, London, Luxembourg, Madrid, and Paris) during the period 2014–2020.

## Methodology and modelization

We use techniques based on long-range dependence or long memory that imply that the infinite sum of the autocovariances is infinite. Within this class of models, widely used in environmental studies, a very manageable one is that based on the concept of fractional integration that means that the number of differences required to render the series stationary and short memory or I(0) may be a fractional real value. According to the definitions provided in Granger (1980, 1981), Granger and Joyeux (1980), and Hosking (1981), a covariance stationary process {*x*(*t*), *t* = 0, ±1, …} is said to be integrated of order *d* and denoted as I(*d*) if it can be expressed as

1 $${\left(1-L\right)}^dx(t)=u(t),\kern1.75em t=1,2,\cdots,$$

where *L* is the lag-operator, that is, *L*^*k*^*x*(*t*) = *x*(*t*-*k*), and *u*(*t*) is I(0) or a short-memory process. Then, if *d* > 0, *x*(*t*) displays long memory (or long-range dependence) in the sense that the observations are highly dependent on time even if they are far distant, and the higher the value of *d* is, the higher the level of association between the observations is.

In the empirical application carried out in the “Empirical Results” section, we allow for deterministic terms such as an intercept and/or a linear time trend. Thus, we consider the following regression model:

2 $$y(t)=\alpha +\beta t+x(t),\kern1.5em t=1,2,\cdots$$

where *y*(*t*) represents the observed data, *α* and *β* are the unknown coefficients, and *x*(*t*) is described by Eq. (1) so that *x*(*t*) is I(*d*). Thus, there are two relevant parameters here: *β* indicating the number of emissions per unit and *d* measuring the degree of persistence in the data.

The estimation is conducted via the Whittle function in the frequency domain by means of implementing a particular version of the tests of Robinson (1994) widely used in empirical applications in the environmental field (Gil-Alana 2009, 2017; Yuan et al. 2013; Gil-Alana and Sauci 2019; etc.).

## Data

The series analysed corresponds to the air quality daily average taken from the World Air Quality Index (WAQI) at https://aqicn.org/map/world/es/. All data have been converted using the United States Environmental Protection Agency (US EPA standard). More specifically, we use data from January 1, 2018, to December 31, 2020, concerning the 20 megacities around the World: Bangkok, Beijing, Bombay, Calcutta, Canton, Dacca, Delhi, Jakarta, London, Los Angeles, Mexico City, Moscow, New York, Osaka, Paris, Sao Paulo, Seoul, Shanghai, Tientsin, and Tokyo. The series represents the daily level of quality in the air based on the measurement of PM_2.5_ microparticles measured in micrograms per cubic meter of air (μg/m^3^). The original sources are Bangkok—Pollution Control Department of Thailand: http://aqmthai.com/web/main.php; Beijing—Beijing Environmental Protection Monitoring Center: https://www.bjmemc.com.cn; Bombay—U.S. Embassy and Consulates’ Air Quality Monitor in India: https://in.usembassy.gov/embassy-consulates/new-delhi/air-quality-data/; Calcutta**—**U.S. Embassy and Consulates’ Air Quality Monitor in India: https://in.usembassy.gov/embassy-consulates/new-delhi/air-quality-data/; Canton**—**Guangdong Environmental Protection public network: www.gdep.gov.cn; Dacca **-** Dhaka Air Quality Monitor—US Consulate: https://bd.usembassy.gov/; Delhi**—**Delhi Pollution Control Committee (Government of NCT of Delhi): http://www.dpccairdata.com/dpccairdata/display/index.php; Jakarta**—**Badan Meteorologi, Klimatologi dan Geofisika (BMKG): https://www.bmkg.go.id/; London**—**London Air Quality Network-Environmental Research Group, King’s College London: https://londonair.org.uk/LondonAir/Default.aspx; Los Angeles**—**South Coast Air Quality Management District (AQMD): http://www.aqmd.gov/; Mexico City**—**National Institute of Ecology and Climate Change (INECC): http://sinaica.inecc.gob.mx/; Moscow**—**Moscow State environmental monitoring: https://mosecom.mos.ru/; New York**—**New York State Department of Environmental Conservation (NYSDEC): https://www.dec.ny.gov/; Osaka**—**Japan Atmospheric Environmental Regional Observation System: http://soramame.taiki.go.jp/; Paris**—**AirParif-Air quality monitoring association in Île-de-France: https://www.airparif.asso.fr/; Sao Paulo**—**CETESB São Paulo State Environmental Company: https://cetesb.sp.gov.br/; Seoul**—**South Air Korea Environment Corporation: https://www.airkorea.or.kr/web; Shanghai—Shanghai Environment Monitoring Center: https://sthj.sh.gov.cn/; Tientsin**—**Tianjin Environmental Monitoring Center: http://www.tjemc.org.cn/html/1/index.html; Tokyo**—**Tokyo, Japan Environment Agency (Tokyo Metropolitan Government Bureau of Environment): https://www.kankyo.metro.tokyo.lg.jp/.

Table 2 shows the number of unavailable observations and their percentage with respect to the series for each city. In these cases, we have computed the arithmetic mean. We observe that the highest percentage of missing observations corresponds to Moscow (21.29%), though they are rather dispersed across the sample, not altering the overall evolution of the data.

**Table 2:** Time series data

City	State	Number of missing observations	Percentage of missing observations
Bangkok	Thailand	2	0.18
Beijing	China	0	0.00
Bombay	India	100	9.14
Calcutta	India	89	8.13
Canton	China	0	0.00
Dacca	Bangladesh	84	7.67
Delhi	India	25	2.28
Jakarta	Indonesia	59	5.39
London	UK	2	0.18
Los Angeles	USA	11	1.00
Mexico	Mexico	204	18.64
Moscow	Russia	233	21.29
New York	USA	30	2.74
Osaka	Japan	7	0.63
Paris	France	2	0.18
Sao Paulo	Brasil	124	11.33
Seoul	Korea	2	0.18
Shanghai	China	2	0.18
Tientsin	China	2	0.18
Tokyo	Japan	7	0.63

The appendix shows the graphs that allow visualizing the behaviour of the concentration of PM_2.5_ in each of the megacities studied. We can observe the elevated levels and variability of some of the megacities, such as Bangkok, Beijing, Bombay, Calcutta, Canton, Dacca, Delhi, Jakarta, Seoul, Shanghai, and Tientsin.

## Empirical results

We consider in this section the model given by Eqs. (1) and (2), testing the null hypothesis

3 $${H}_o:d={d}_o,$$

for any real value *d*_*o*_. Thus, the model under the null becomes

4 $$y(t)=\alpha +\beta t+x(t);\kern1em {\left(1-L\right)}^{d_o}x(t)=u(t).\kern1.5em t=1,2,\cdots$$

Across the tables, we report the values of *d*_*o*_ where the null hypothesis (3) cannot be rejected at the 95% level along with the estimates of *d* based on a frequency domain version of the Whittle function. We display the estimates of *d* under three different scenarios: (i) no deterministic terms, i.e. implying that *α* = *β* = 0 a priori in (4), (ii) with an intercept or a constant (i.e. with *β* = 0 a priori), and (iii) with both the constant and the linear time trend freely estimated from the data.

In Tables 3 and 4, we suppose *u*(*t*) is a white noise process, while in Tables 5 and 6, weakly autocorrelated errors are permitted. We start by reporting the results based on white noise errors. We first notice that the time trend is not required in any single case, and the estimated values of *d* are constrained between 0 and 1 in all cases and thus showing fractional integration. However, the values of *d* substantially change from one series to another. Thus, there are two cities where the estimated values of *d* are found to be significantly below 0.5 and thus showing a stationary pattern. They are Shanghai (*d* = 0.40) and New York (0.44). On the other hand, there are nine cities with *d* displaying non-rejection values below and above 0.5. They are Tientsin (0.43), Beijing and Tokyo (0.46), Canton (0.52), Mexico (0.54), Osaka and Sao Paulo (0.46), and Moscow (0.56). Finally, the remaining ten cities show nonstationary patterns, with values of *d* significantly above 0.50 and ranging from Jakarta (*d* = 0.54) to Bangkok (*d* = 0.79). Nevertheless, in all cases, the estimated values of *d* for all cities are significantly smaller than 1 showing mean reversion and transitory shocks, disappearing thus in the long run. Table 3 displays the estimated coefficients, the lowest intercept corresponds to the case of Tokyo (40.632), and the largest one referring to Delhi (332.621).

**Table 3:** Estimates of the differencing parameter. White noise errors

City	No deterministic terms	An intercept	An intercept with a linear time trend
Bangkok	0.81 (0.75, 0.88)	**0.79 (0.73, 0.87)**	0.79 (0.73, 0.87)
Beijing	0.48 (0.41, 0.56)	**0.46 (0.38, 0.54)**	0.46 (0.38, 0.54)
Bombay	0.75 (0.70, 0.80)	**0.68 (0.63, 0.74)**	0.68 (0.63, 0.74)
Calcutta	0.78 (0.73, 0.84)	**0.74 (0.69, 0.80)**	0.74 (0.69, 0.80)
Canton	0.56 (0.52, 0.62)	**0.52 (0.47, 0.59)**	0.53 (0.47, 0.59)
Dacca	0.71 (0.66, 0.76)	**0.64 (0.60, 0.70)**	0.65 (0.60, 0.70)
Delhi	0.64 (0.60, 0.70)	**0.62 (0.57, 0.67)**	0.62 (0.57, 0.68)
Jakarta	0.56 (0.52, 0.61)	**0.54 (0.50, 0.60)**	0.55 (0.50, 0.60)
London	0.65 (0.59, 0.72)	**0.60 (0.53, 0.69)**	0.60 (0.53, 0.69)
Los Angeles	0.66 (0.59, 0.73)	**0.68 (0.60, 0.76)**	0.68 (0.61, 0.76)
Mexico	0.59 (0.53, 0.65)	**0.54 (0.48, 0.61)**	0.54 (0.48, 0.61)
Moscow	0.59 (0.53, 0.66)	**0.56 (0.49, 0.64)**	0.56 (0.49, 0.64)
New York	0.50 (0.45, 0.57)	**0.44 (0.39, 0.51)**	0.44 (0.39, 0.51)
Osaka	0.59 (0.53, 0.67)	**0.55 (0.47, 0.64)**	0.55 (0.47, 0.64)
Paris	0.68 (0.61, 0.75)	**0.65 (0.57, 0.73)**	0.65 (0.57, 0.73)
Sao Paulo	0.58 (0.51, 0.66)	**0.55 (0.48, 0.64)**	0.55 (0.48, 0.64)
Seoul	0.62 (0.55, 0.71)	**0.60 (0.53, 0.70)**	0.60 (0.53, 0.70)
Shanghai	0.47 (0.42, 0.53)	**0.40 (0.34, 0.47)**	0.40 (0.34, 0.47)
Tientsin	0.46 (0.40, 0.54)	**0.43 (0.35, 0.52)**	0.43 (0.35, 0.52)
Tokyo	0.50 (0.45, 0.56)	**0.46 (0.39, 0.53)**	0.45 (0.39, 0.53)

The values in bold refer to the selected specification in relation to the deterministic terms. The values in parenthesis are the 95% confidence bands of the non-rejection values of *d*

**Table 4:** Estimated coefficients from Table 3

City	*d* (95% confidence band)	Intercept (*t*-value)	Time trend (*t*-value)
Bangkok	0.79 (0.73, 0.87)	98.971 (6.88)	-
Beijing	0.46 (0.38, 0.54)	97.607 (4.76)	-
Bombay	0.68 (0.63, 0.74)	205.698 (11.58)	-
Calcutta	0.74 (0.69, 0.80)	211.03 (8.29)	-
Canton	0.52 (0.47, 0.59)	120.498 (8.08)	-
Dacca	0.64 (0.60, 0.70)	213.736 (9.45)	-
Delhi	0.62 (0.57, 0.67)	332.621 (9.48)	-
Jakarta	0.54 (0.50, 0.60)	64.921 (5.24)	-
London	0.60 (0.53, 0.69)	56.731 (5.17)	-
Los Angeles	0.68 (0.60, 0.76)	115.581 (9.83)	-
Mexico	0.54 (0.48, 0.61)	68.926 (7.43)	-
Moscow	0.56 (0.49, 0.64)	42.860 (4.28)	-
New York	0.44 (0.39, 0.51)	42.773 (7.96)	-
Osaka	0.55 (0.47, 0.64)	64.913 (5.89)	-
Paris	0.65 (0.57, 0.73)	43.819 (3.74)	-
Sao Paulo	0.55 (0.48, 0.64)	45.506 (3.74)	-
Seoul	0.60 (0.53, 0.70)	85.704 (4.25)	-
Shanghai	0.40 (0.34, 0.47)	107.875 (9.01)	-
Tientsin	0.43 (0.35, 0.52)	111.804 (6.32)	-
Tokyo	0.46 (0.39, 0.53)	40.632 (6.15)	-

**Table 5:** Estimates of the differencing parameter. Autocorrelated errors

City	No deterministic terms	An intercept	An intercept with a linear time trend
Bangkok	0.59 (0.53, 0.67)	**0.51 (0.45, 0.58)**	0.51 (0.45, 0.58)
Beijing	0.15 (0.08, 0.25)	0.10 (0.04, 0.16)	**0.08 (0.02, 0.15)**
Bombay	0.65 (0.60, 0.72)	0.55 (0.51, 0.61)	**0.56 (0.51, 0.62)**
Calcutta	0.64 (0.60, 0.70)	**0.58 (0.54, 0.64)**	0.58 (0.54, 0.64)
Canton	0.45 (0.39, 0.51)	0.36 (0.30, 0.43)	**0.36 (0.30, 0.42)**
Dacca	0.62 (0.57, 0.67)	**0.54 (0.50, 0.59)**	0.54 (0.50, 0.59)
Delhi	0.55 (0.50, 0.61)	0.50 (0.45, 0.56)	**0.51 (0.46, 0.58)**
Jakarta	0.48 (0.44, 0.54)	**0.45 (0.40, 0.50)**	0.45 (0.41, 0.51)
London	0.42 (0.36, 0.47)	0.26 (0.21, 0.32)	**0.20 (0.12, 0.28)**
Los Angeles	0.33 (0.27, 0.40)	**0.27 (0.22, 0.34)**	0.28 (0.21, 0.36)
Mexico	0.45 (0.40, 0.52)	**0.34 (0.29, 0.42)**	0.34 (0.28, 0.42)
Moscow	0.43 (0.36, 0.52)	**0.32 (0.23, 0.42)**	0.32 (0.22, 0.42)
New York	0.32 (0.28, 0.39)	0.23 (0.18, 0.29)	**0.22 (0.17, 0.28)**
Osaka	0.34 (0.29, 0.41)	0.16 (0.10, 0.22)	**0.11 (0.04, 0.20)**
Paris	0.40 (0.35, 0.47)	0.26 (0.20, 0.35)	**0.23 (0.15, 0.32)**
Sao Paulo	0.30 (0.25, 0.36)	**0.23 (0.19, 0.29)**	0.23 (0.18, 0.29)
Seoul	0.28 (0.23, 0.33)	0.21 (0.16, 0.26)	**0.20 (0.15, 0.25)**
Shanghai	0.31 (0.25, 0.37)	0.19 (0.12, 0.23)	**0.14 (0.09, 0.21)**
Tientsin	0.12 (0.04, 0.21)	**0.06 (0.02, 0.12)**	0.06 (0.01, 0.12)
Tokyo	0.30 (0.25, 0.36)	0.17 (0.12, 0.22)	**0.11 (0.05, 0.18)**

The values in bold refer to the selected specification in relation to the deterministic terms. The values in parenthesis are the 95% confidence bands of the non-rejection values of *d*

**Table 6:** Estimated coefficients from Table 5

City	*d* (95% confidence band)	Intercept (*t*-value)	Time trend (*t*-value)
Bangkok	0.51 (0.45, 0.58)	99.354 (11.78)	-
Beijing	0.08 (0.02, 0.15)	114.400 (29.36)	−0.0166 (−2.76)
Bombay	0.56 (0.51, 0.62)	195.057 (13.26)	−0.0783 (−1.93)
Calcutta	0.58 (0.54, 0.64)	208.193 (10.74)	-
Canton	0.36 (0.30, 0.42)	109.094 (12.04)	−0.0280 (−1.90)
Dacca	0.54 (0.50, 0.59)	204.969 (11.60)	-
Delhi	0.51 (0.46, 0.58)	295.826 (10.39)	−0.1163 (−1.86)
Jakarta	0.45 (0.40, 0.50)	77.061 (9.04)	-
London	0.20 (0.12, 0.28)	67.950 (26.81)	−0.0228 (−5.90)
Los Angeles	0.27 (0.22, 0.34)	55.837 (23.09)	-
Mexico	0.34 (0.29, 0.42)	65.425 (17.14)	-
Moscow	0.32 (0.23, 0.42)	50.416 (14.35)	-
New York	0.22 (0.17, 0.28)	40.519 (16.83)	−0.0076 (−2.03)
Osaka	0.11 (0.04, 0.20)	73.983 (41.38)	−0.0152 (−5.50)
Paris	0.23 (0.15, 0.32)	66.340 (23.31)	−0.0158 (−3.64)
Sao Paulo	0.23 (0.19, 0.29)	55.035 (22.44)	-
Seoul	0.20 (0.15, 0.25)	90.419 (21.19)	−0.0191 (−2.95)
Shanghai	0.14 (0.09, 0.21)	109.094 (12.04)	−0.0268 (−4.10)
Tientsin	0.06 (0.01, 0.12)	204.969 (11.60)	-
Tokyo	0.11 (0.05, 0.18)	50.849 (34.07)	−0.0153 (−6.68)

In Tables 5 and 6, we allow for weak autocorrelation. We employ here a Bloomfield exponential spectral model (Bloomfield 1973) that accommodates autoregressive (AR) structures in a nonparametric way with a lower number of parameters, being stationary for all its range of values unlike what happens in the AR case.

We observe in Table 5 that the time trend is required now in a number of cases (11 out of the 20 cases presented), and the time trend coefficient is negative in all these cases, the largest value corresponding to Delhi (−0.1163), followed by Bombay (−0.0783). This is good news in the sense that it indicates a systematic decrease in the number of emissions for these cities. The estimates of d are once again smaller than 1 but the values are now much lower. Stationary patterns (*d* < 0.50) are observed in 15 out of the 20 observed series, ranging the values of d from 0.06 (Tientsin) to 0.45 (Jakarta); for Bangkok, Dacca, and Delhi, the estimates of *d* are around 0.50, and Bombay and Calcutta display values of *d* significantly higher than 0.50 and thus showing a nonstationary pattern. Nevertheless, in all cases, the estimated values of *d* are once again more statistically significantly below 1 and thus displaying mean reversion and implying transitory shocks. Thus, in the event of a negative exogenous shock generating an increase in the emissions in the cities, there is no need for strong actions since the series will return by themselves in the long run to the original long-term projection. On the other hand, if the shock is positive, reducing substantially the number of emissions, actions should be conducted to maintain the emissions at these lower levels.

Table 7 summarizes the results in terms of the degree of persistence measured by the fractional differencing parameter, *d*. The left column refers to the case of white noise errors, while the right-hand column refers to autocorrelation. If we look at the first five positions (referring to the lowest degrees of persistence), we observe that there are three cities appearing in both scenarios: Shanghai (1st position with white noise errors and 5th under autocorrelation), Tientsin (2nd and 1st respectively) and Tokyo (5th and 3rd). On the other hand, focusing on the bottom places with the highest levels of persistence, three cities share the last 4 places: Bangkok (20th and 17th), Calcutta (19th and 20th), and Bombay (18th and 19th). The lowest values observed under the assumption of autocorrelation can be explained by the competition between the two structures (fractional differentiation and Bloomfield autocorrelation) in describing the time dependence. Note that under the white noise structure, all the time dependence is exclusively captured by the order of integration d, while in the autocorrelation case, the degree of integration competes with the Bloomfield structure in capturing that time lag relationship.

**Table 7:** Summary results on persistence

White noise		Autocorrelation	
Shanghai	0.40 (0.34, 0.47)	Tientsin	0.06 (0.01, 0.12)
Tientsin	0.43 (0.35, 0.52)	Beijing	0.08 (0.02, 0.15)
New York	0.44 (0.39, 0.51)	Tokyo	0.11 (0.04, 0.20)
Beijing	0.46 (0.38, 0.54)	Osaka	0.11 (0.05, 0.18)
Tokyo	0.46 (0.39, 0.53)	Shanghai	0.14 (0.09, 0.21)
Canton	0.52 (0.47, 0.59)	London	0.20 (0.12, 0.28)
Mexico	0.54 (0.48, 0.61)	Seoul	0.20 (0.15, 0.25)
Jakarta	0.54 (0.50, 0.60)	New York	0.22 (0.17, 0.28)
Osaka	0.55 (0.47, 0.64)	Paris	0.23 (0.15, 0.32)
Sao Paulo	0.55 (0.48, 0.64)	Sao Paulo	0.23 (0.19, 0.29)
Moscow	0.56 (0.49, 0.64)	Los Angeles	0.27 (0.22, 0.34)
London	0.60 (0.53, 0.69)	Moscow	0.32 (0.23, 0.42)
Seoul	0.60 (0.53, 0.70)	Mexico	0.34 (0.29, 0.42)
Delhi	0.62 (0.57, 0.67)	Canton	0.36 (0.30, 0.42)
Dacca	0.64 (0.60, 0.70)	Jakarta	0.45 (0.40, 0.50)
Paris	0.65 (0.57, 0.73)	Delhi	0.51 (0.46, 0.58)
Los Angeles	0.68 (0.60, 0.76)	Bangkok	0.51 (0.45, 0.58)
Bombay	0.68 (0.63, 0.74)	Dacca	0.54 (0.30, 0.59)
Calcutta	0.74 (0.69, 0.80)	Bombay	0.56 (0.51, 0.62)
Bangkok	0.79 (0.73, 0.87)	Calcutta	0.58 (0.54, 0.64)

It is also interesting to see at the time trend coefficients, which are significantly negative in a number of cases under the hypothesis of autocorrelated errors: Delhi (−0.116), Bombay (−0.0783), Canton (−0.0280), London (−0.0228), Shanghai (−0.0226), Seoul (−0.0191), Beijing (−0.0166), Paris (−0.0158), Tokyo (−0.0153)**,** Osaka (−0.0152), and New York (-0.0076). For the rest of the cities (Bangkok, Calcutta, Dacca, Jakarta, Los Angeles, Mexico, Moscow, Sao Paulo, and Tientsin), the time trend coefficient is found to be statistically insignificantly different from zero.

In Table 8, we compare the time trend coefficients under the (wrong) assumption of I(0) errors, i.e. imposing a priori that *d* = 0, (2nd column) with those obtained with *d* estimated from the data (3rd column). We observe that for seven cities, namely, Bangkok, Calcutta, Dacca, Mexico, Moscow, Sao Paulo, and Tientsin, the time trend coefficient is significant when *d* = 0 but insignificant when *d* is correctly estimated. For a couple of cities, Jakarta and Los Angeles, the coefficient is insignificant in the two scenarios, and for the remaining eleven cases, there is a reduction in the magnitude of *β* in six cities (Beijing, London, Osaka, Paris, Seoul, and Tokyo) and an increase in another five (Bombay, Canton, Delhi, New York, and Shanghai). Thus, erroneous conclusions can be obtained under the imposition of short memory or I(0) structures on the error term.

**Table 8:** Summary results on time trends

City	*d* = 0	*d* estimated
Bangkok	−0.0184 (−11.12)	-
Beijing	−0.0172 (−3.60)	−0.0166 (−2.76)
Bombay	−0.0578 (−19.77)	−0.0783 (−1.93)
Calcutta	−0.0560 (−14.48)	-
Canton	−0.0198 (−7.96)	−0.0280 (−1.90)
Dacca	−0.0413 (−10.96)	-
Delhi	−0.0515 (−9.69)	−0.1163 (−1.86)
Jakarta	-	-
London	−0.0252 (−17.72)	−0.0228 (−5.90)
Los Angeles	-	-
Mexico	−0.0166 (−8.37)	-
Moscow	−0.0080 (−3.86)	-
New York	−0.0064 (−5.11)	−0.0076 (−2.03)
Osaka	−0.0155 (−9.84)	−0.0152 (−5.50)
Paris	−0.0187 (−13.74)	−0.0158 (−3.64)
Sao Paulo	−0.0096 (−4.57)	-
Seoul	−0.0203 (−8.18)	−0.0191 (−2.95)
Shanghai	−0.0264 (−8.22)	−0.0268 (−4.10)
Tientsin	−0.0085 (−2.18)	-
Tokyo	−0.0163 (−11.71)	−0.0153 (−6.30)

## Conclusions

In this paper, we have looked at the statistical properties of PM_2.5_ in 20 megacities around the world. Using daily data from January 1, 2018, to December 31, 2020, and based on fractional integration, our results show that the estimated values for the differencing parameter are constrained in the interval (0, 1) in all cases, thus showing a mean reverting pattern and thus implying transitory effects of shocks. This result holds independently of the way of modelling the I(0) error term either with uncorrelation or with autocorrelated errors, though lower degrees of integration are shown under this latter assumption. More importantly, the fact that the differencing parameter is significatively different from zero has some important consequences on the estimation of the time trend coefficient that measures the decrease in the emissions per unit. In fact, some megacities that were supposed to display significant negative time trends under the I(0) specification are found to show insignificant coefficients with the long-memory approach. This is one important lesson obtained in this work. Thus, when examining time trends in environmental series the potential presence of long memory is an issue that should be taken into account.

Finally, this work can be extended in several directions. First, the same type of analysis can be extended to other regions of the world in order to verify if the long-memory property holds across regions and to check the decrease in the values across time. Second, other long-memory models, based on parametric, semiparametric, or even nonparametric methods can be implemented to verify the results reported in this work. Finally, the possibility of nonlinear structures or structural changes still in the context of fractional integration in the analysis of environmental data is another line of future research that is currently in progress.

## Data Availability

The datasets generated during and/or analysed during the current study are available from the corresponding author on reasonable request.

## Appendix

Time series plots of PM_2.5_ measurements between 2018 and 2020 for the 20 megacities

The abscissa axis shows the monthly average from 2018 to 2020, and the ordinate axis shows the measurements of PM_2.5_ microparticles measured in micrograms per cubic meter of air (μg/m^3^).
